# Mature Twin Neonates Exhibit Oxidative Stress via Nitric Oxide Synthase Dysfunctionality: A Prognostic Stress Marker in the Red Blood Cells and Umbilical Cord Vessels

**DOI:** 10.3390/antiox9090845

**Published:** 2020-09-10

**Authors:** Payal Chakraborty, Krisztina N. Dugmonits, Hajnalka Orvos, Edit Hermesz

**Affiliations:** 1 Department of Biochemistry and Molecular Biology, Faculty of Science and Informatics, University of Szeged, P.O.Box 533, H-6701 Szeged, Hungary; payal96.pharm@gmail.com (P.C.); krisztina.dugmonits@gmail.com (K.N.D.); 2 Department of Obstetrics and Gynaecology, Faculty of Medicine, University of Szeged, P.O.Box 533, H-6701 Szeged, Hungary; orvosh@gmail.com

**Keywords:** arginase, birthweight, blood, endothelial dysfunction, nitric oxide synthase, twin pregnancy, umbilical cord

## Abstract

Intrauterine hypoxic condition increases the generation of reactive oxygen species and fetal oxidative stress. Multiple pregnancy always bears an additional oxidative stress condition with severe complications, such as prematurity, structural abnormalities, delayed development and low birthweight. The umbilical cord (UC) vessels, along with circulating fetal red blood cells (RBCs), highly determine the oxygenation status of fetus and regulate the feto-placental circulation. As UC lacks innervation, the activation of the endothelial nitric oxide synthase (NOS3) is fundamental for proper NO production. Therefore, we aimed to study the NOS3 activation pathways along with damages to macromolecules in the endothelium of UC vessels and RBCs of mature non-discordant twins, in connection to major differences in their birth weight. We provide evidence that, under severe hypoxic conditions such as twin pregnancy, the NOS3-related NO production pathways are altered both in UC vessels and RBCs; moreover, the extent of changes is highly birthweight-specific. Furthermore, macromolecular damages are prominent in the RBCs and arteries compared to the vein, with a similar increase in the Arginase1 level, which is believed to play a role in NOS3 functionality, resulting in endothelial dysfunctionality, which might have relevance to the major etiologies of cardiovascular diseases in later life.

## 1. Introduction

Increasing evidence suggests that enhanced oxidative insults during intrauterine development frequently contribute to the occurrence of pathological conditions in post-natal or adult life [1,2]. With considerable alterations in the physiological and metabolic functions, the delicate balance between reactive oxygen species (ROS), and the intrinsic antioxidant defense system is disrupted. The process of pregnancy deals with redox homeostasis imbalance due to increased ROS level, which gets aggravated during twinning or multiple pregnancy, and might cause indiscriminate damage to biological molecules, leading to loss of function and even cell death [3]. Twins are also more prone to birth asphyxia, hyaline membrane disease, respiratory disorders and long-term developmental morbidity [4,5,6,7].

During pregnancy, the placenta and the umbilical cord form vital temporary organs to sustain in utero conditions, and therefore, most complications are directly or indirectly associated with placental or umbilical cord disorders, such as intrauterine hypoxia and an impaired blood flow to the fetus [8,9,10]. The umbilical cord lacks innervation, thus, the vascular tone and integrity majorly depend on the cord endothelial-derived nitric oxide (NO) via the functional nitric oxide synthase (NOS3) [11,12]. The subcellular localization of NOS3 and its activation pathway are tightly controlled by major factors such as the substrate L-arginine concentration, availability of co-factors, rate of electron transfer, post-translational modifications and diverse interacting proteins. These factors can be highly affected by excessive ROS production with low endogenous antioxidant capacity possibly influenced by intrinsic or extrinsic pathophysiological factors, leading to oxidative stress conditions [13]. The endothelial NOS3 activation pathway is mainly calcium-dependent via the PI3-Akt pathway. NOS3 gets phosphorylated at serine1177 residue to produce bioavailable and vasoactive NO [14,15]. Recent studies also cited the expression of Arginase isoforms in the blood vessels, mainly from the vascular endothelium, which have a common substrate with NOS3 and L-arginine, and play critical roles in the regulation of L-arginine homeostasis and the production of L-ornithine for subsequent polyamine and proline synthesis. Polyamine and proline are the key components for the physiological and pathological vascular remodeling, endothelial and smooth muscle cell proliferation and collagen deposition [16]. In addition, due to increased expression of Arginase the available concentration of L-arginine substrate gets lowered, which indirectly influences the NOS3 coupling process. As a result, the NOS3 protein remains uncoupled in their inactive monomer state, which produces an excess of superoxide anion (O_2_^•−^) instead of NO. The elevated level of O_2_^•−^ undergoes a spontaneous reaction and scavenges the limited bioavailable NO in the vascular system by forming deleterious pro-oxidant radical peroxynitrite (ONOO^−^) [17,18,19].

The vascular endothelium comes in direct contact with all the blood components and shares an especially intimate crosstalk with the predominant circulating red blood cells (RBCs). For a long time, RBCs were taken as carriers for tissue oxygenation, transmission of metabolic gases and nutrients and sink for NO. The added ground-breaking facts on the human circulatory RBCs with the ‘erythrocrine function’ under physiological condition defines a new functional role of RBCs. It had been well stated by Kelm et al. that adult RBCs are not only passive regulators of the endothelium-derived NO level, but the RBCs themselves are able to synthesize and export NO, promoting the maintenance of the vascular tone and blood flow [20,21,22]. Furthermore, Pernow et al. published clear evidence in adults that, under disease states, RBCs show excess levels of ROS, altered protein profile and have an intimate crosstalk with the vascular endothelium and synergizes the process of vascular impairment, termed ‘erythropathy’ [23,24].

Previously, our working group demonstrated an increased ROS production and compromised antioxidant system with an increased level of NOS3, leading to elevated macromolecular damage in the fetal RBCs derived from twin neonates [25]. In continuation of this project and citing the intimate crosstalk in between the vascular endothelium and the circulating RBCs, we further elucidated the NOS3 regulation and activity in detail on the fetal RBCs from cord artery and endothelial cells of the umbilical cord derived from twin neonates and singletons. We hypothesize that any molecular changes and the antioxidant status of the cord vein may indicate the quality of the “nutrition” coming from the mother, while the states of the arteries provide more information concerning the actual state of the fetus.

## 2. Methods and Materials

### 2.1. Human Samples

In compliance with the principles as outlined in the Declaration of Helsinki, we took pregnant mothers’ informed consent and collected a part of the umbilical cord along with fetal cord blood samples from the Department of Obstetrics and Gynecology at the University of Szeged, Hungary. The Ethics Committee of the Department approved the study protocol (16/2014; Investigation of oxidative stress markers in maternal and neonatal blood samples). According to our study protocol for sample collection, these exclusion factors were strictly followed: (i) maternal age below 18 years, (ii) gestational age less than 37 weeks, (iii) gestational diabetes, infection and inflammatory conditions or disorders such as cardiovascular diseases, (iv) complications or difficulty during delivery, (v) fetal distress, (vi) malformations or evidence of genetic disorders and (vii) neonates from mothers addicted to alcohol and smoking habit. The nutritional status of the mothers during pregnancy was satisfactory; no case of malnutrition occurred. In total, 21 pairs of mature non-discordant twin neonates and 32 age- and weight-matched singletons were taken as controls in comparison. Twin neonates were categorized based on their birth weight; 3000–3200 gr (High birthweight twin—Hwt) and 2400–2600 gr (Low birthweight twin—Lwt) in comparison to age- and weight-matched singletons as High birthweight singleton (Hws) and Low birthweight singleton (Lws). The clinical parameters of the study groups and the maternal age are presented in the Table 1.

### 2.2. Sample Processing for Immunostaining

Fragments of UCs were fixed in 4% (*w/v*) paraformaldehyde in 0.05 M phosphate buffer pH 7.4 (PB) and cryo-protected with 30% (*w/v*) sucrose in PB, supplemented with 0.1% (*w/v*) Na-azide. Specimens were embedded in Tissue-Tek^®^ O.C.T.™ (Sakura Europe 4583, Alphen aan den Rijn, Netherlands), cryosectioned (16 µm), mounted on Superfrost™ ultra plus^®^ (Thermo scientific J3800AMNZ, Massachusetts, United States) microscope slides and kept at –80 °C until further processed [26]. Whole blood was centrifuged at 200× *g* for 10 min at 4 °C, only the lower two-thirds of the RBC phase was collected, this was fixed at 4% (*w/v*) paraformaldehyde in 0.05 M PB at 4 °C for 1 h and processed for immunolabelling [27].

### 2.3. Immunolabelling on Umbilical Cord and Cord Blood Samples

Immunostaining was carried out on thawed slides and fixed RBCs. After permeabilization by 0.1% Triton X-100 at room temperature for 20 min, non-specific antibody binding sites were blocked with 4% (*w/v*) bovine serum albumin (BSA) and 5% (*v/v*) normal goat serum in PB. Samples were immunolabelled with primary antibodies overnight at 4 °C. Incubations with the primary antibodies was followed by washing and incubation with goat anti-mouse/anti-rabbit Alexa^®^647- or goat anti-rabbit/anti-mouse Alexa^®^488-conjugated secondary antibodies in 1:2000 dilution, for 2 h in the dark at room temperature (for the detailed antibody list see supplementary information Table S1). After incubation slides were consecutively washed and counterstained with 4′,6-diamidino-2-phenylindole (DAPI) (D9542) from Sigma-Aldrich (St. Louis, MO, USA) with a final concentration of 1 µg/mL, for 5 min in the dark, dried and mounted in Antifading, BrightMount/Plus aqueous mounting medium (Abcam ab103748, Cambridge, United Kingdom) and examined under epifluorescence microscope (Nikon Eclipse 80i, 100× and 50× immersion objective; Nikon Zeiss Microscopy GmbH, Jena, Germany); pictures were taken with a QImaging RETIGA 4000R camera, using Capture Pro 6.0 software (QImaging, Surrey, BC, Canada). During imaging, arteries and veins of each samples were captured at 5–10 independent fields, resulting in at least 50 individual images of each vessel circumference. Images were analyzed by ImageJ^®^ software [28]. In brief, composite pictures were split into channels, each channel corresponded to single specific antibody labelling. The endothelial layer was marked out by an indicator software tool selection, and the relevant signal particles were analyzed by running a pre-recorded macro, which was optimized to our study protocol. The parameters were kept constant throughout the evaluation protocol. As a result, we got and saved the relevant regions of interest (ROI), and as it projected to our original images, the mean grey values were measured, which were proportional to the fluorescent intensities. These values were corrected to their background fluorescence by marking out and quantifying five independent non-antibody specific fluorescent areas in each image.

RBCs, after incubation with secondary antibodies, were washed and processed for quantitative analysis (FACS (Fluorescence-activated cell sorting), BD FACSCalibur™, BD Biosciences, New Jersey, United States) [28,29]. The homogeneity and purity of the isolated RBCs were tested by staining with RBC-specific marker anti Glycophorin A. Purity of the samples was >95% for RBCs. In case of viability assay isolated RBCs were 10× diluted in 0.9% (*w/v*) physiological salt solution having pH 7.4, and 1 µL of it was analyzed using AnnexinV-FITC (Fluorescein IsoThioCyanate) Apoptosis/Staining Detection kit (Abcam ab14085, Cambridge, United Kingdom). Briefly, 1 µL diluted RBCs were mixed with 250 µL of Annexin binding buffer and 2.5 µL AnnexinV which were subjected for quantitative analysis (FACS, BD FACSCalibur™, BD Biosciences) [30]. All FACS data were analyzed using FlowJo™ (FlowJo™ Software for Windows Version 10, Oregon, United States).

### 2.4. Statistical Analysis

Statistical calculations were done by one-way analysis of variance (ANOVA) and Newman-Keuls multiple comparison test using GraphPad Statistical Software version 6.0 (GraphPad Software, San Diego, CA, USA). All statistically significant differences were accepted at ∗ *p* ≤ 0.05, ∗∗ *p* ≤ 0.01, ∗∗∗ *p* ≤ 0.001 and ∗∗∗∗ *p* ≤ 0.0001.

## 3. Results

### 3.1. NOS3 Expression and Its Activation Level Is Altered Both in the Umbilical Cord Vessels and in the Isolated RBCs

Under severe oxidative stress condition, the bioavailability of NO via NOS3 activation pathway is pivotal in the maintenance of the vasodilatory mechanism. NOS3 expression and its posttranslational modification in the vascular cord endothelium along with the isolated RBC population taken from twin and singleton neonates were followed.

**Figure 1:**
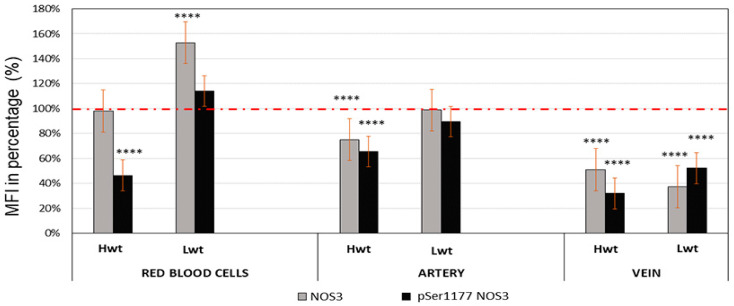
Quantification of the NOS3 expression and its activation level at pSer1177 residue. Mean fluorescent intensity (MFI) were expressed in the percentage of the subsequent High birthweight twin (Hwt) and Low birthweight twin (Lwt) population in comparison to their age and weight matched singletons High birthweight singleton (Hws) and Low birthweight singleton (Lws) as controls. The red dotted line represents the control intensity level at 100% within the both umbilical cord vessel endothelium and isolated fetal arterial cord red blood cells (RBCs). **** Marks the significant differences based on the Newman-Keuls multiple comparison test at *p* < 0.0001.

Anti-NOS3/pSer1177 NOS3 double-labelled sections of the umbilical cords originated from twins and singletons were subjected to Image J© evaluation to follow NOS3 expression and its phosphorylation status (pSer1177 NOS3). In general, the NOS3 and the pSer1177 NOS3 levels were significantly lower in the endothelium of both the arteries and veins, originated from Hwt, as compared to the age and weight matched controls. The NOS3 lowered by 25% and 50%, while the pSer1177 NOS3 by ~35% and 69%, in the artery and vein, respectively. In the Lwt group, the NOS3 and pSer1177 NOS3 level was significantly lower in the vein endothelium, with only ~37% and 52% of the matching control value, while in the artery there was no significant changes in either value. Based on the ratio of NOS3/pSer1177 NOS3 values, the NOS3 phosphorylation status remained more or less unchanged, except the Lwt vein endothelium, where the phosphorylation status slightly increased (Figure 1, Figures S1–S3)

**Figure 2:**
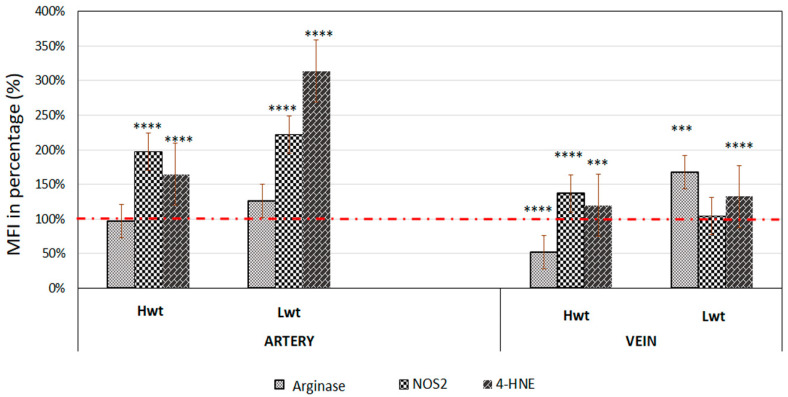
Quantification of the expression level of Arginase1, inducible NOS (NOS2) and the extent of macromolecular damages as followed with the lipid peroxidation marker 4-HNE. Mean fluorescent intensity (MFI) was expressed in percentage of the mature twin neonates (Hwt and Lwt) with their age and weight matched singletons (Hws and Lws) taken as controls. The intensity level of the controls was considered as 100% (marked with red dotted line). The significant differences were accepted at *** *p* < 0.001 and **** *p* < 0.0001 based on one-way ANOVA using the Newman-Keuls multiple comparison test.

In stress conditions, inducible nitric oxide synthase (NOS2) might be an alternative NO producing pathway. Umbilical cord sections were immunolabelled with anti-NOS2 and evaluated by Image J©. The arterial endothelium represented significant rise in the NOS2 level with 98% in Hwt and 122% in Lwt groups. In the vein endothelium of Hwt, there was a moderate increase of 37%, while NOS2 level stayed unchanged in the Lwt group (Figure 2, Figures S4–S6).

**Figure 3:**
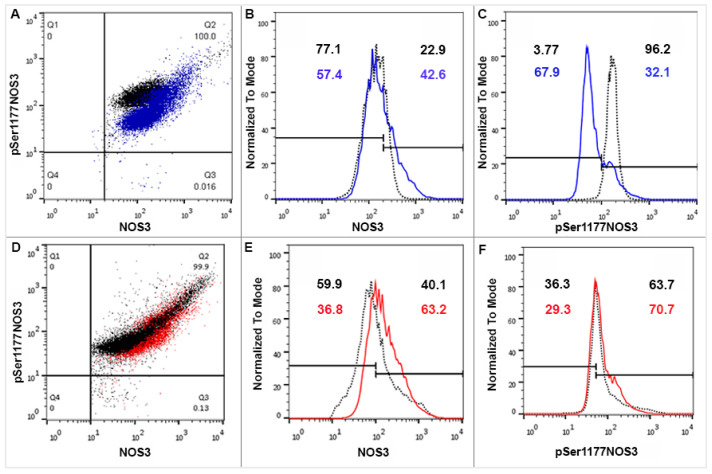
FACS (Fluorescence-activated cell sorting) analysis on arterial cord RBCs immunolabelled with NOS3 and pSer1177 NOS3 from the mature twin neonates and their matched singletons. Representative panels (**A**,**D**) show the dot plots of Hwt vs Hws and Lwt vs Lws RBCs, immunolabelled with anti-NOS3 and anti-pSer1177 NOS3 primary antibodies, respectively. The histograms (**B**,**E**) using FACS analysis show the NOS3 intensities in the Hwt (blue line) and Lwt (red line) with their matched singletons (black dots), similarly histograms (**C**,**F**) exhibit pSer1177 NOS3 intensities; in each case, according to the blank, an arbitrary borderline was considered dividing the total RBC population into basal and high intensities.

In the isolated RBCs, NOS3 and pSer1177 NOS3 levels underwent a FACS analysis. In case of the Hwt group, NOS3 was almost identical with the singletons, while in the Lwt category, there was a significant, ~53% increase measured. The pSer1177 NOS3 level highly lagged behind the control (by ~54%) in the Hwt, while a moderate increase (~14%) was measured in the Lwt group (Figure 1). In the detailed FACS analysis, the total NOS3 expression was divided into basal and high levels. In each case, according to the blank sample, an arbitrary borderline was considered along the x-axis, dividing the total RBC population into basal and high intensity groups (Figure 3). Based on this division, it is clear that the frequency of the high NOS3 expressing cells increased both in the Lw and Hw twin groups by 48% and 60%, respectively. However, it does not mean an increase in the NOS3 activation, since its phosphorylation status is much lower (ratios of NOS3/pSer1177 NOS3) than that measured in Hw and Lw singletons.

**Figure 4:**
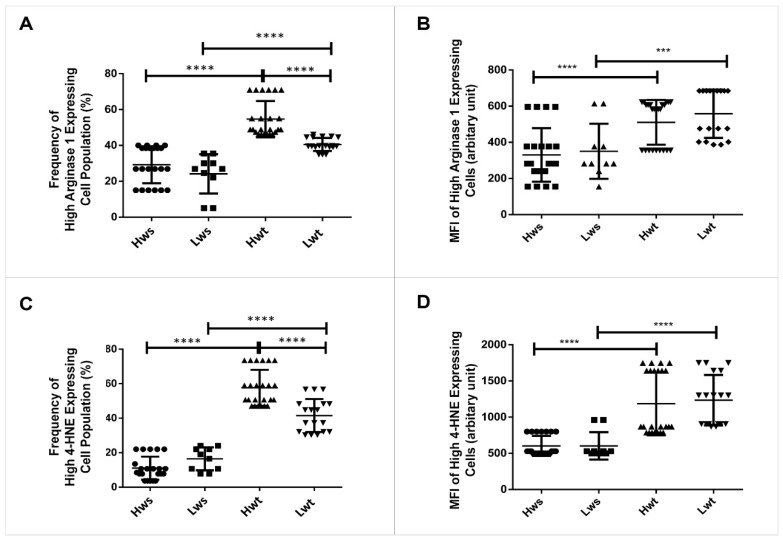
Measurement of the frequency and MFI (mean fluorescent intensity) of high-expressing Arginase1 and 4-HNE in the arterial cord RBCs. The graphical representation in the panels (**A**,**B**) and (**C**,**D**) indicate the frequency and MFI of high expressing Arginase1 and 4-HNE RBC population in the mature twin neonates compared to their matched singletons. The statistical significance was accepted at *** *p* < 0.001 and **** *p* < 0.0001 based on one-way ANOVA using the Newman-Keuls multiple comparison test.

NOS3 competes with Arginase1 for their common substrate L-arginine. Umbilical cord artery and vein endothelium immunolabelled with Arginase1 were evaluated by Image J©. There lies a significant difference between the vein and arterial endothelium in Hwt and Lwt groups. In the Hw twins’ artery, the Arginase1 intensity level was in close match with the singleton, whereas it is decreased by 48% in the vein. In contrast, the Lwt group showed an increase by 26% in the artery and 68% in the vein (Figure 2, Figures S4–S6). In the isolated RBCs, the Arginase1 level underwent a FACS analysis. In general, Arginase1 expression increased in both the Hw and Lw weight twin groups. Moreover, there was a significant increase in the frequency of high Arginase1 expressing cells population, irrespective of the difference in their birth weight; 86% in the Hwt and 67% in the Lwt group (Figure 4A,B, Figure S7A,B).

### 3.2. Quantitative Measurement of Lipid Peroxidation Marked with 4-Hydroxynonenal and Annexin V Positive Levels in Twin Pregnancy

The most common products of lipid peroxidation, due to excessive ROS production, are the oxygenated α, β-unsaturated aldehydes such as 4-hydroxynonenal (4-HNE). The formation of this aldehyde was followed by immunolabelling the aldehyde-protein adduct with anti-4-HNE antibody. Using Image J© to measure the 4-HNE intensity level in the cord endothelium, there showed a significant increase in the artery of both high and low weight twin groups, 65% and 214%, respectively. The vein endothelium demonstrated only a moderate increase of 20% in Hw and 33% in Lw twins (Figure 2, Figures S4–S6).

In the RBC population, the 4-HNE intensity level and Annexin V positivity underwent FACS analysis. The result showed a prominent increase in the 4-HNE intensity level both in the Hwt and Lwt groups. In the detailed FACS analysis, in each case, according to the blank sample, we divided the total RBC population into basal and high intensity groups by an arbitrary borderline along the x-axis. There is a higher frequency with an ~422% and 153% increase in the high and low weight twin group, respectively, in the high 4-HNE expressing cells, which ultimately results in a total higher intensity level of 4-HNE in the twin neonates compared to the singletons (Figure 4C,D, Figure S7C,D).

**Figure 5:**
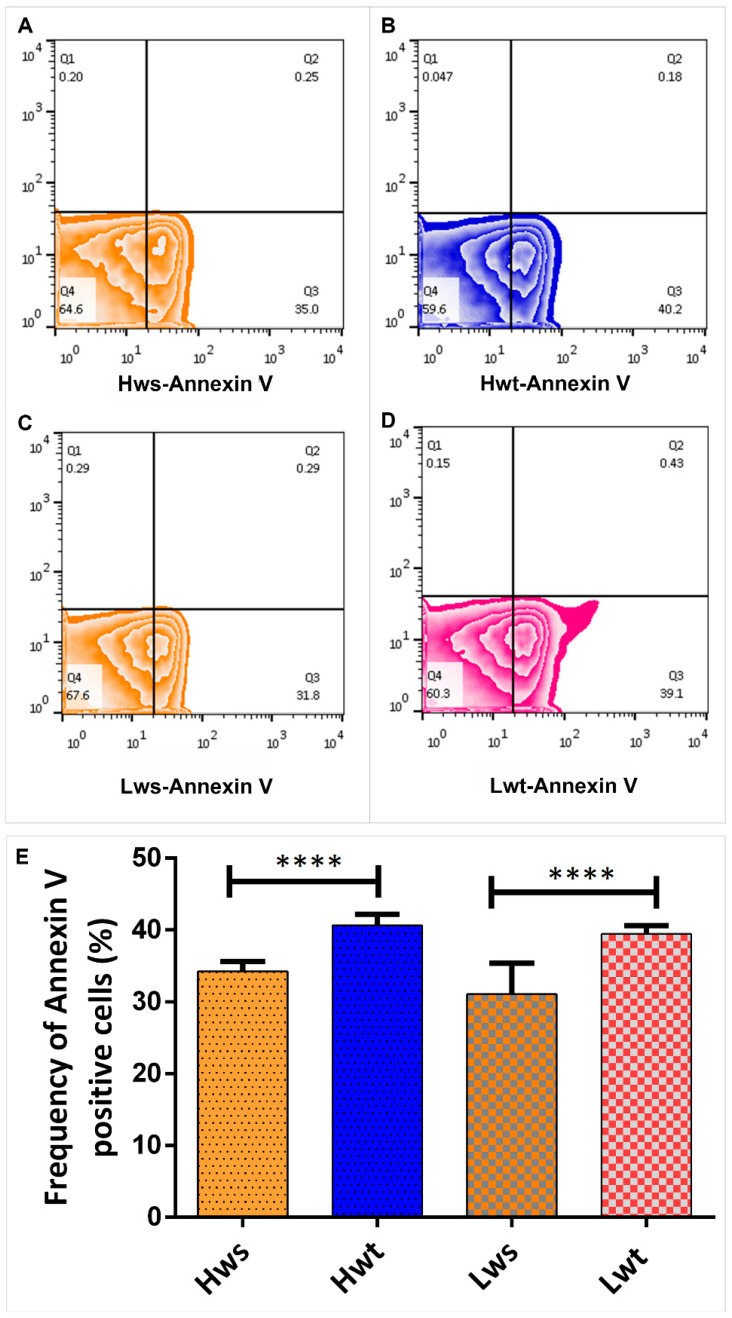
Viability Assay on isolated RBCs by the FITC (Fluorescein IsoThioCyanate) -conjugated Annexin V. Panel (**A**–**D**) are representative zebra plots derived from isolated RBCs of the age and weight matched Hws, Hwt, Lws and Lwt, respectively. Based on the blank value of the appropriate control and twin sample, an arbitrary borderline was considered along the x-axis, where the Q3 quadrant represents the AnnexinV positive cells. The graphical summary (**E**) shows the percentage distribution of Annexin V positive RBCs localized in Q3 quadrant. Statistical significance was accepted at **** *p* < 0.0001 using the Newman-Keuls multiple comparison test.

In parallel to 4-HNE, the neonatal RBCs from both singleton and twins were stained by Annexin V, which detects and quantify the level of phosphatidylserine in the outer leaflet of RBC plasma membranes. The frequency of Annexin-V positive cells in the twin neonatal RBCs in all cases were significantly higher, by 27% in the Lwt and 19% in the Hwt, compared to the matching singletons (Figure 5A–E).

## 4. Discussion

Intrauterine pre-placental hypoxia commonly occurs due to multiple pregnancy, manifesting different outcomes in the maternal and fetal conditions. A state of pre-placental hypoxia primarily depends on intrinsic placental intervillous development with connection to local oxygen conditions [31,32]. Due to multiple pregnancy, the uterus gets highly distended, having a decreased oxygen pressure in the environment. This alters the intra-placental oxygen content that affects the maturation in the utero-placental intervillous exchange surface, causing low materno-fetal oxygen transfer [33]. Thus, in the case of twin/multiple pregnancy, there exists a chronic state of maternal hypoxemia with a reduced oxygen content of the blood passing through the placenta. In that situation, NO plays an exceptionally crucial role in the process of vasorelaxation and sufficient blood supply to the fetus. In our previous study, we demonstrated an elevated NOS3 expressing population of RBCs in twin neonates compared to singletons [25]. Based on the data by Pernow et al. [23,24], in the present study we looked for a cross-talk between circulating RBCs and vascular endothelium and focused on the detailed molecular mechanism of NO production not only limited to the RBC population, but also in the UC endothelium. We demonstrated, for the first time, that due to twin pregnancy (1) the NOS3 expression/activation pathway are impaired both in the RBCs and in the endothelium of UC vessels, but clearly at different level of regulation. Moreover, the extent of the damage is somewhat in correlation with the birthweight in all the three constituents of the feto-vascular system (RBC, artery and vein). The NOS3 level in the arterial and venous cord endothelium showed a prominent and a mild dropdown in the Hw and Lw twin groups, respectively, without significant changes in the level of NOS3 phosphorylation at serine1177 residue, whereas in the RBC populations the NOS3 level either stay unchanged or somewhat elevated, while its phosphorylation lagged behind extremely. Our results also indicate that (2) because of the insufficient NO production by NOS3, an alternative NO producing pathway, upregulation of NOS2, could serve as compensatory mechanism. Finally, (3) Arginase1 expression gets highly upregulated in the twin RBC population and elevation in the Arginase1 expression occurs through a ROS dependent mechanism, which causes an increased rate of lipid peroxidation, as marked by the 4-HNE level and Annexin V with loss in the RBC membrane properties and its functionality.

There are multiple pathways that can influence the NOS3 activation. Primarily, the NOS3 dimer formation and its phosphorylation at serine1177 residue are crucial criteria in the functional activation of NOS3 [17]. In accordance with our previous findings and recent publications, endogenous or the acquired exogenous antioxidant defense mechanisms are not fully functional, as the expression of genes coding for antioxidants are downregulated in the twin neonates with comparison to singletons [25]. On this background, the homeostatic balance between the prooxidant and antioxidant gets easily disrupted and the presence of excess ROS highly influence and regulate the NOS3 activation pathway by the induction of Arginase1 expression. The NOS3 activation pathway gets interlinked with Arginase1 expression which competes for the common substrate L-arginine. Arginase1 reciprocally inhibits the NOS3 expression by “stealing away” the common substrate L-arginine, required to generate bioavailable NO and L-citrulline and instead produces excess of O_2_^•−^ in the circulatory system [13,14,18,19,34]. The limited amount of bioavailable NO in the system gets further scavenged by the O_2_^•−^ forming the deleterious prooxidant like ONOO^−^ showing the “Janus faced” role of NO. An alternative NO producing pathway, the NOS2 is highly upregulated in all type of vessel group but the Lwt vein. Though the increased NOS2 expression seems beneficial providing bioavailable NO, the boosted NO production by NOS2, due to its high (100–1000 fold that of the NOS3) catalytic activity, might also lead to ROS elevation and induced cell apoptosis through caspase-3 activation [35,36]. Indeed, the level of macromolecular damages aggravates in the cord artery of twin neonates, where the NOS2 level is by far the highest in the system. Following the RBCs, the membrane damage also significantly increased in both the low and high weight twin neonates, signifying the greater extent of lipid peroxidation. This result is further supported by the significant increase in the Annexin V positive cells implying to altered RBC membrane asymmetry, membrane damage and loss of functional properties in the neonatal RBCs of twins.

## 5. Conclusions

An understanding of the basic pathophysiological mechanisms in neonatal diseases necessitates a detailed knowledge about the wide range of complications in both the circulating fetal RBCs along with the umbilical cord endothelium. In this study we demonstrated that the endothelium in both the UC vessels of neonates born from twin pregnancy lose their characteristic functional features with a greater impact on the arterial cord endothelium. Due to such a high degree of arterial endothelial impairment, there is an upregulation of the compensatory/rescue mechanism in the system. Based on our result it seems that NOS2 might be a valuable/promising marker to characterize this mechanism. Similar to the vessel endothelium, the NOS3-NO production pathway in the RBCs is also impaired. Here, the main pivotal fact is the increased level of Arginase1 regulating the complex mechanism of NOS3 uncoupling. Due to the intimate crosstalk in between the RBCs and the vascular endothelium, the Arginase level could serve as a real time prognostic marker not only for RBCs, but to track the overall changes in the vascular microenvironment.

## Figures and Tables

**Table 1:** The clinical parameters of the study groups and the maternal age.

Clinical Parameters	Full-Term Single Neonates	Full-Term Twin Neonates
Numbers of Samples (*N*)	Hws: *N* = 22 Lws: *N* = 10	Hwt: *N* = 24 Lwt: *N* = 18
Gestational age at delivery (weeks)	37.52 ± 0.50 (37–38.6)	37.35 ± 0.47 (37–38.4)
Birth weight (kg)	Hws: 3.30 ± 0.172 (3.0–3.5) Lws: 2.59 ± 0.171 (2.49–2.8)	Hwt: 3.19 ± 0.073 (3.0–3.3) Lwt: 2.6 ± 0.174 (2.2–2.9)
APGAR score at 10 min	9.92 ± 0.27 (9–10)	9.69 ± 0.53 (8–10)
Ratio of vaginal delivery/caesarean section	28:4	34:8
Maternal age (years)	29.5 ± 6.11 (20–42)	31.9 ± 7.12 (24–40)
Blood sample pH	7.26 ± 0.13 (7.03–7.42)	7.29 ± 0.12 (7.19–7.44)

## Supplementary Materials

Supplementary materials are available online at https://www.mdpi.com/2076-3921/9/9/845/s1.
