# Supplementary material for: Mature Twin Neonates Exhibit Oxidative Stress via Nitric Oxide Synthase Dysfunctionality: A Prognostic Stress Marker in the Red Blood Cells and Umbilical Cord Vessels
Source: Antioxidants (Basel). 2020 Sep 10;9(9):845. doi: 10.3390/antiox9090845 (PMC7555925; doi:10.3390/antiox9090845)
Supplement: Supplementary file 1 [file antioxidants-09-00845-s001.pdf]

Supporting Information

Title: Mature twin neonates exhibit oxidative stress via nitric oxide synthase dysfunctionality: A prognostic stress marker in the red blood cells and umbilical cord vessels

Payal Chakraborty, Krisztina N. Dugmonits, Hajnalka Orvos, Edit Hermesz.

Table of Contents

Table S1. List of the sources and dilutions of primary and secondary antibodies. 2

Figure S1. The representative epifluorescent images of the arterial endothelium in relation to NOS3 expression and its phosphorylation level. 3

Figure S2. Representative epifluorescent images of the venous endothelium in relation to the NOS3 expression and its phosphorylation level. 4

Figure S3. Illustration of the MFI (mean florescence intensities) of NOS3 expression and its phosphorylated level (pNOS3) in the both RBCs and Vascular Endothelium. 5

Figure S4. Represents epifluorescent images of the arterial cord endothelium sections immunolabelled with the Arginase1, NOS2 and 4-HNE. 6

Figure S5. Visualization of the vein endothelium sections epifluorescent images immunolabelled with the Arginase1, NOS2 and 4-HNE. 7

Figure S6. Illustration of the MFI (mean florescence intensities) of Arginase1, NOS2 expression and subsequent extent of lipid peroxidation by 4-HNE level in the Vascular Endothelium. 8

Figure S7. Evaluation of the immunolabelled Arginase1 and 4-HNE level on the arterial cord RBCs by FACS analysis. 9

Table S1. List of the sources and dilutions of primary and secondary antibodies.

Antibody	Host	Dilution	Code	Distributor
anti-Glycophorin A	mouse	1:50	MA5-12484	Thermo Fisher Scientific, Madison, WI, USA
anti-NOS3	mouse	1:100	sc-376751	Santa Cruz Biotechnology Inc., Dallas, TX, USA
anti-pSer1177 NOS3	rabbit	1:100	SAB-4300128	Sigma Aldrich, Saint Louis, Missouri, USA
anti-Arginase1	mouse	1:100	sc-166920	Santa Cruz Biotechnology Inc., Dallas, TX, USA
anti-NOS2	rabbit	1:100	ab3523	Abcam, Cambridge, UK
anti 4-hydroxy-2-nonenal (4-HNE)	mouse	1:100	ab48506	Abcam, Cambridge, UK
goat anti-mouse Alexa®647	mouse	1:400	ab150115	Abcam, Cambridge, UK
goat anti-rabbit Alexa®488	rabbit	1:400	ab150077	Abcam, Cambridge, UK
goat anti-mouse Alexa®488	mouse	1:2000	ab150113	Abcam, Cambridge, UK
goat anti-rabbit Alexa®647	rabbit	1:2000	ab150079	Abcam, Cambridge, UK

Figure S1. The representative epifluorescent images of the arterial endothelium in relation to NOS3 expression and its phosphorylation level.

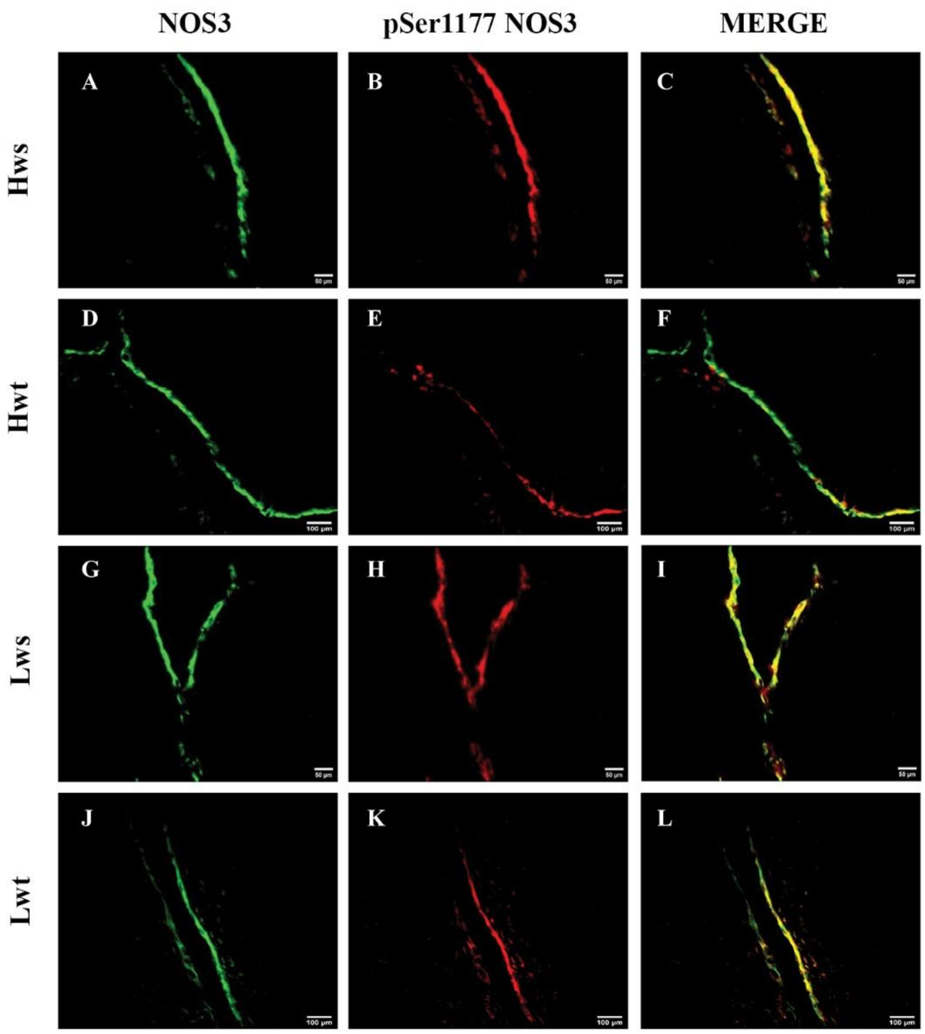

Figure S1. Representative epifluorescent images of immunolabelled arterial endothelium originated from Hws, Hwt, Lws and Lwt samples. Panels (A, D, G and J) show immunolabelling with mouse primary anti-NOS3 antibody followed by an Alexa Fluor® 488 secondary antibody. Panels (B, E, H and K) show immunolabelling with a rabbit anti-pSer1177 NOS3 primary antibody followed by an Alexa Fluor® 647 secondary antibody. Panels (C, F, I and L) present the merged images. Slides were mounted and examined under an epifluorescence microscope (Nikon Eclipse 80i, 100x and 50x immersion objective; Nikon Zeiss Microscopy GmbH, Jena, Germany).

Figure S2. Representative epifluorescent images of the venous endothelium in relation to the NOS3 expression and its phosphorylation level.

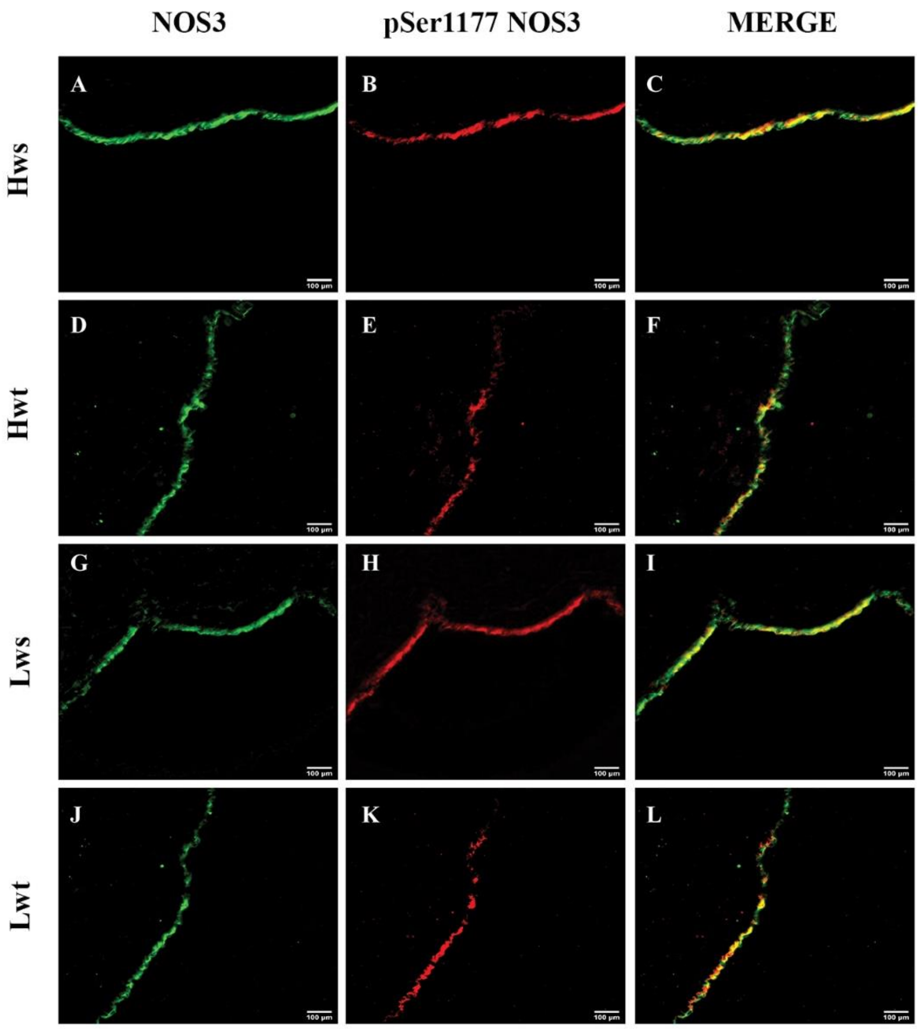

Figure S2. Representative epifluorescent images of immunolabelled venous endothelium originated from Hws, Hwt, Lws and Lwt samples. Panels (A, D, G and J) show immunolabelling with mouse primary anti-NOS3 antibody followed by an Alexa Fluor® 488 secondary antibody. Panels (B, E, H and K) show immunolabelling with a rabbit anti-pSer1177 NOS3 primary antibody followed by an Alexa Fluor® 647 secondary antibody. Panels (C, F, I and L) present the merged images. Slides were mounted and examined under an epifluorescence microscope (Nikon Eclipse 80i, 100x and 50x immersion objective; Nikon Zeiss Microscopy GmbH, Jena, Germany). (Nikon Eclipse 80i, 100x and 50x immersion objective; Nikon Zeiss Microscopy GmbH, Jena, Germany).

Figure S3. Illustration of the MFI (mean florescence intensities) of NOS3 expression and its phosphorylated level (pNOS3) in the both RBCs and Vascular Endothelium.

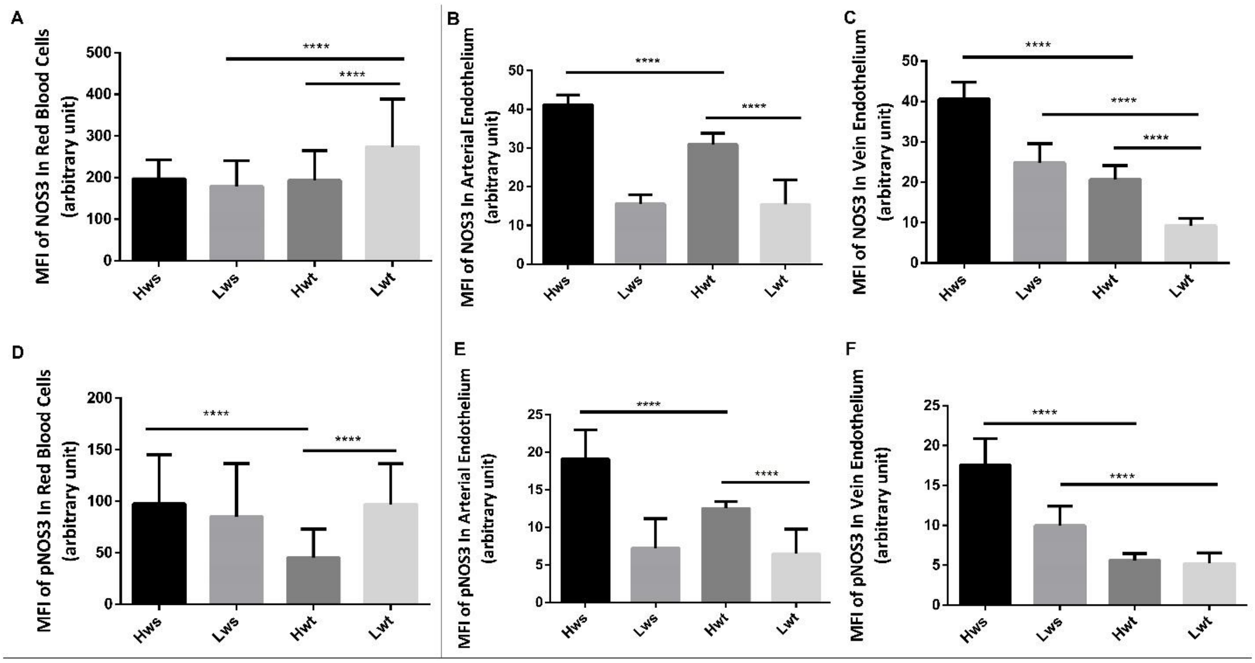

Figure S3. Graphically illustrate the MFI quantification of NOS3 expression panels (A-C) and its activation/phosphorylated level (pNOS3) at Ser1177 residue panel (D-F) in the RBCs, arterial endothelium and vein endothelium respectively, derived from both Hw and Lw twin groups with their matched singletons (Hws and Lws). The significant differences were accepted at ****p<0.0001 based on one-way ANOVA using the Newman-Keuls multiple comparison test.

Figure S4. Represents epifluorescent images of the arterial cord endothelium sections immunolabelled with the Arginase1, NOS2 and 4-HNE.

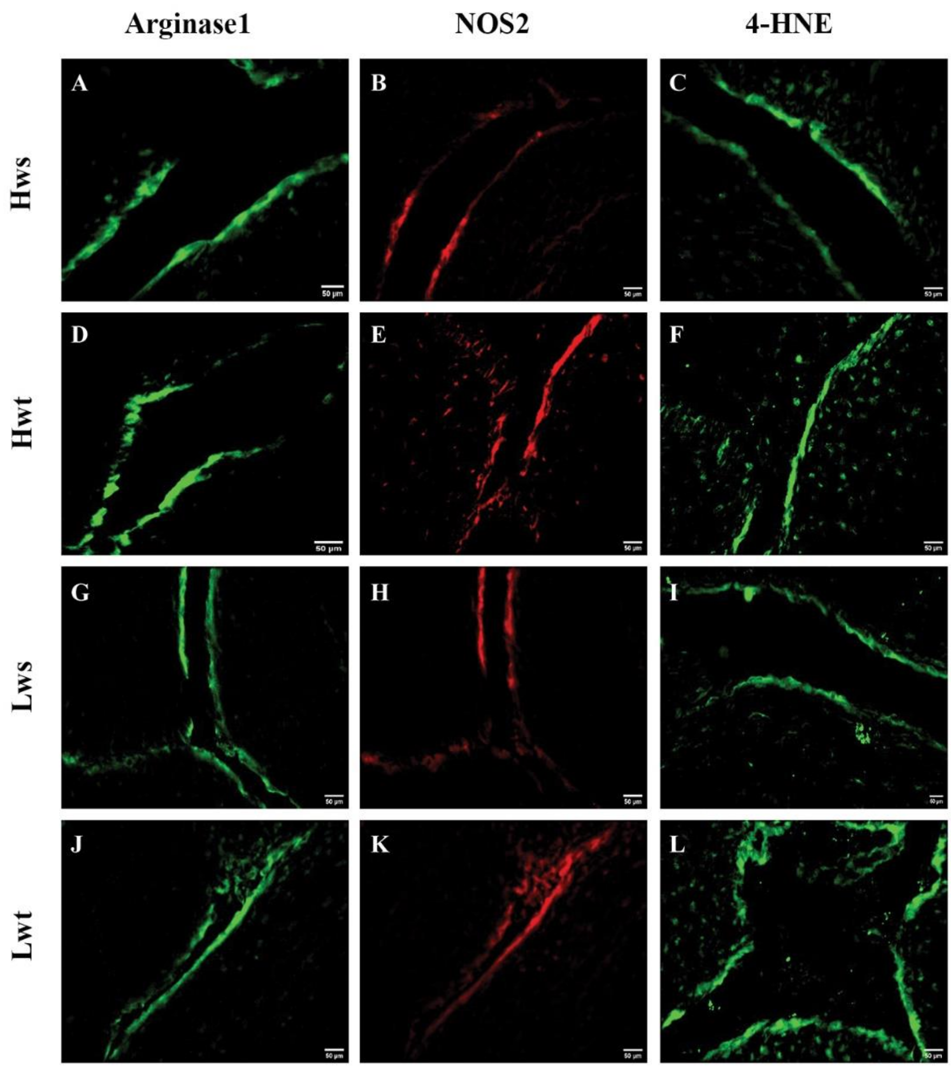

Figure S4. Representative epifluorescent images of immunolabelled arterial endothelium originated from Hws, Hwt, Lws and Lwt samples. The panels (A, D, G and J) show immunolabelling with a mouse primary anti-Arginase1 antibody followed by an Alexa Fluor® 488 secondary antibody. In the similar manner, panels (B, E, H and K) present samples immunolabelling with a rabbit primary anti-NOS2 antibody followed by an Alexa Fluor® 647 secondary antibody and the panels (C, F, I and L) exhibit immunolabelling with a mouse primary anti 4-HNE antibody followed by an Alexa Fluor® 488 secondary antibody. The slides were mounted and examined under an epifluorescence microscope (Nikon Eclipse 80i, 100x and 50x immersion objective; Nikon Zeiss Microscopy GmbH, Jena, Germany).

Figure S5. Visualization of the vein endothelium sections epifluorescent images immunolabelled with the Arginase1, NOS2 and 4-HNE.

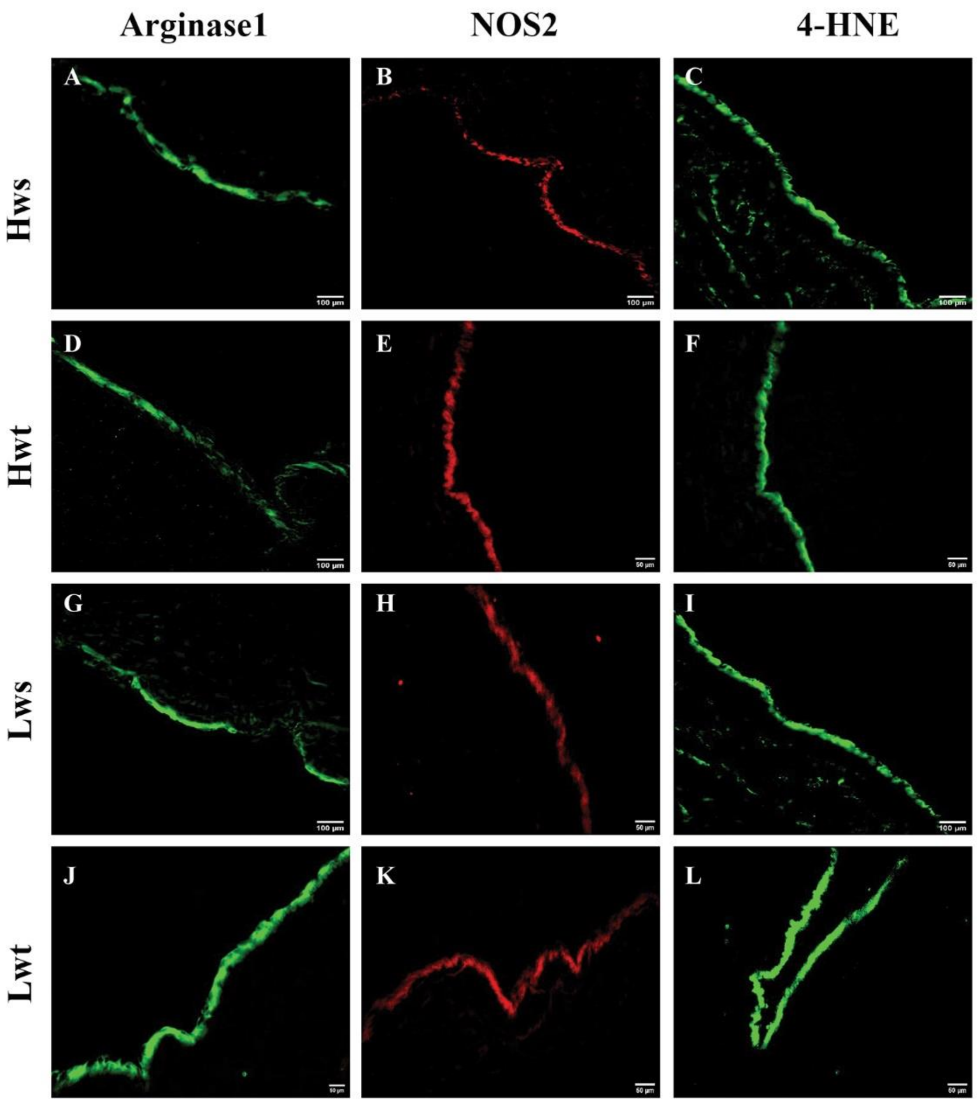

Figure S5. Representative epifluorescent images of immunolabelled venous endothelium originated from Hws, Hwt, Lws and Lwt samples. The panels (A, D, G and J) show immunolabelling with a mouse primary anti-Arginase1 antibody followed by an Alexa Fluor® 488 secondary antibody. In the similar manner, panels (B, E, H and K) present samples immunolabelling with a rabbit primary anti-NOS2 antibody followed by an Alexa Fluor® 647 secondary antibody and the panels (C, F, I and L) exhibit immunolabelling with a mouse primary anti 4-HNE antibody followed by an Alexa Fluor® 488 secondary antibody. The slides were mounted and examined under an epifluorescence microscope (Nikon Eclipse 80i, 100x and 50x immersion objective; Nikon Zeiss Microscopy GmbH, Jena, Germany).

Figure S6. Illustration of the MFI (mean florescence intensities) of Arginase1, NOS2 expression and subsequent extent of lipid peroxidation by 4-HNE level in the Vascular Endothelium.

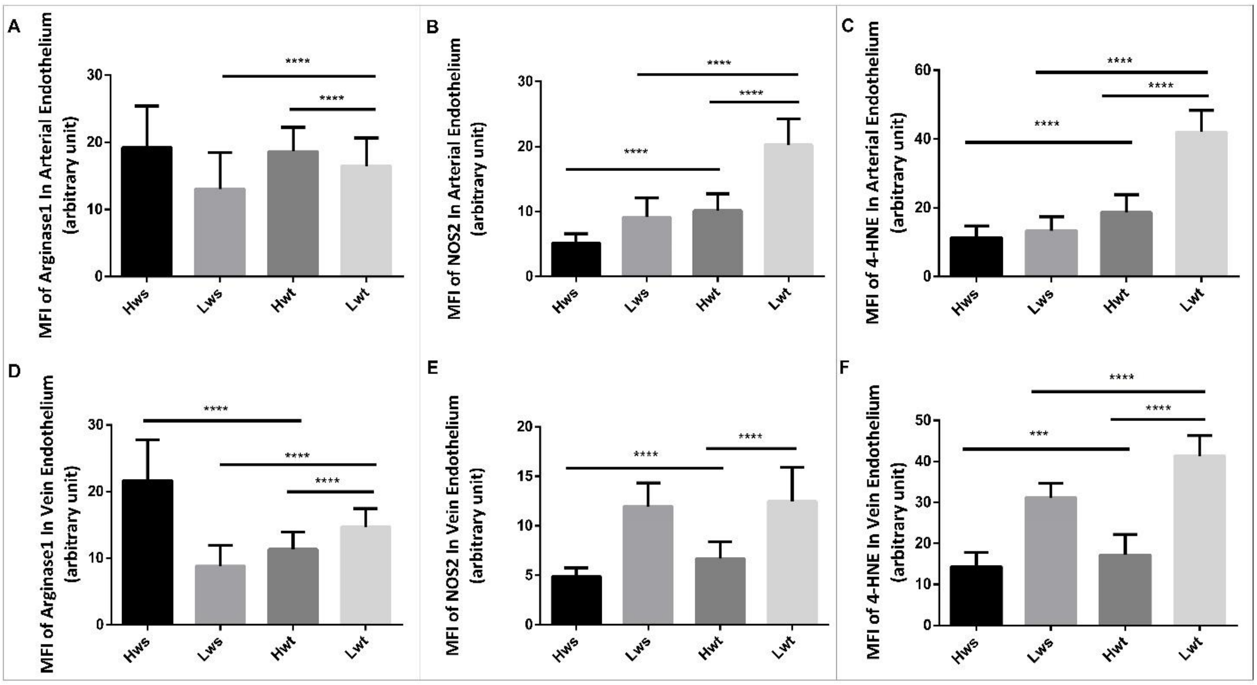

Figure S6. Graphically illustrate the MFI quantification of Arginase1 panels (A and D), NOS2 panels (B and E) expression and the extent of lipid peroxidation by the 4-HNE level panels (C and F) in the both arterial and vein endothelium respectively, derived from both the twin groups (Hwt and Lwt) with their matched singletons (Hws and Lws). The significant differences were accepted at ***p<0.001 and ****p<0.0001 based on one-way ANOVA using the Newman-Keuls multiple comparison test.

Figure S7. Evaluation of the immunolabelled Arginase1 and 4-HNE level on the arterial cord RBCs by FACS analysis.

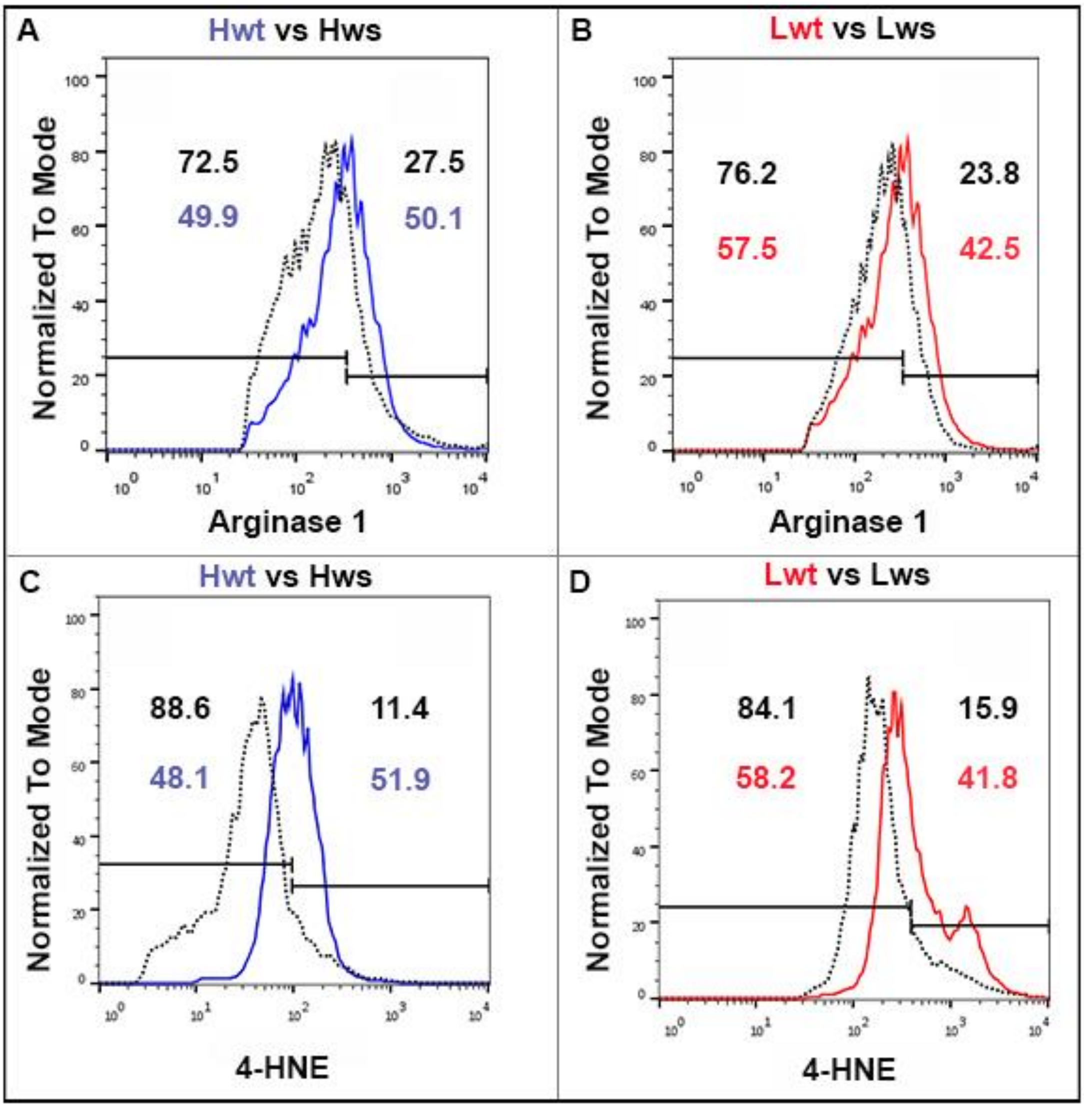

Figure S7. Representative histogram plot as measured by the FACS analysis using anti-Arginase1 panel (A and B) and anti 4-HNE panel (C and D) primary antibody on the fetal arterial cord RBCs derived from Hwt vs Hws and Lwt vs Lws respectively. Based on the blank value for each control and twin sample an arbitrary borderline was considered along the x-axis dividing the total RBC population into basal and high expressing cells.
